# Functional Characterization of Myelodysplastic Syndrome by Multiparameter Flow Cytometry: The Clinical Synergy Between Ki-67 and Bcl-2 and Their Potential Role in Diagnostics and Personalized Therapy

**DOI:** 10.3390/cancers18162648

**Published:** 2026-08-17

**Authors:** Sixuan J. Wang, Rinaldo A. J. N. van Meel, Stefan G. C. Mestrum, Thomas H. P. M. Habets, Anton H. N. Hopman, Frans C. S. Ramaekers, Yvonne M. C. Henskens, Otto Bekers, Mathie P. G. Leers

**Affiliations:** 1 Department of Clinical Chemistry & Hematology, Zuyderland Medical Center, 6162 BG Sittard-Geleen, The Netherlands; s.wang@zuyderland.nl (S.J.W.); r.vanmeel@zuyderland.nl (R.A.J.N.v.M.); stefan.mestrum@outlook.com (S.G.C.M.); 2 NUTRIM-Institute for Nutrition and Translational Research in Metabolism, Maastricht University Medical Center, 6229 HX Maastricht, The Netherlands; 3 Central Diagnostic Laboratory (CDL), Maastricht University Medical Center, 6229 HX Maastricht, The Netherlands; yvonne.henskens@mumc.nl (Y.M.C.H.); o.bekers@mumc.nl (O.B.); 4 Department of Biotechnology, CHILL b.v., 6167 RD Sittard-Geleen, The Netherlands; 5 Department of Genetics & Cell Biology, GROW Research Institute for Oncology and Reproduction, Maastricht University Medical Center, 6229 HX Maastricht, The Netherlands; hopman@maastrichtuniversity.nl (A.H.N.H.); f.ramaekers@maastrichtuniversity.nl (F.C.S.R.); 6 Department of Radiation Oncology (Maastro), GROW Research Institute for Oncology and Reproduction, Maastricht University Medical Center, 6229 HX Maastricht, The Netherlands; thomas.habets@maastro.nl; 7 Department of Environmental Sciences, Faculty of Science, Open Universiteit, 6419 AT Heerlen, The Netherlands

**Keywords:** myelodysplastic syndrome, multiparameter flow cytometry, Ki-67, Bcl-2, Venetoclax, proliferation, apoptosis, precision medicine

## Abstract

The diagnosis and clinical management of myelodysplastic neoplasms are increasingly challenged by the disease’s inherent heterogeneity, particularly with respect to the diagnosis of low-grade variants. While standardized flow cytometric protocols traditionally rely on static biomarkers for lineage assignment, these often fail to capture the dynamic biological behavior of the malignant clone. This review synthesizes studies on the integration of functional biomarkers, specifically the nuclear proliferation marker Ki-67 and the anti-apoptotic protein Bcl-2, into the diagnostic and prognostic workflow. By utilizing high-dimensional multiparameter flow cytometry (MFC) and software-based maturation continuum analysis, the survival and growth kinetics of the myeloid, erythroid, and monocytic lineages can be quantified. These findings redefine myelodysplastic syndromes (MDS) as characterized by a significant decrease in cell-cycle progression and an increase in anti-apoptotic activity during early stages of maturation. Recent studies demonstrate that integrating the erythroid Ki-67 proliferation index as a fifth parameter into the conventional Ogata score dramatically improves diagnostic sensitivity for detecting MDS from 66% to 90% while maintaining 100% specificity. In particular, the sensitivity for detecting low-grade MDS improved from 56% to 91%. Additionally, a reduced erythroid Ki-67 index (≤28%) is a powerful independent predictor of transfusion dependence within 1 year. Beyond diagnostics, the introduction of the Bcl-2:Ki-67 ratio provides a superior metric for biological aggressiveness and a potential predictive tool for precision medicine. A high ratio identifies a quiescent, apoptosis-resistant cell population that is likely refractory to standard chemotherapy but is an ideal candidate for targeted Bcl-2 inhibition with Venetoclax. The integration of functional biomarkers bridges the gap between complex mutational landscapes and clinical manifestations. While digital imaging and artificial intelligence (AI) are beginning to automate blast enumeration and maturation analysis, functional kinetics may provide a necessary biological readout for personalized therapy.

## Simple Summary

The diagnostic landscape of myelodysplastic neoplasms is rapidly evolving, moving toward an integrated framework of morphology, immunophenotyping, cytogenetics, and molecular profiling as defined by the WHO 2022 and International Consensus Classifications (ICC). The diagnosis and clinical management of myelodysplastic syndromes (MDS) are, however, increasingly challenged by the disease’s inherent heterogeneity, particularly within the diagnostic gray zone of low-grade variants. While multiparameter flow cytometry is established for detecting immunophenotypic aberrancies, standard protocols, including those from EuroFlow, often demonstrate limited sensitivity in low-grade MDS. These protocols have traditionally relied on static biomarkers for lineage assignment, but such phenotypic markers often fail to capture the dynamic biological behavior of the malignant clone. By focusing primarily on the cellular phenotype, current diagnostic frameworks often miss the underlying functional kinetics, specifically the critical imbalance between proliferation and apoptosis, that drives ineffective hematopoiesis. This omission means that the subtle yet defining survival and growth advantages of the clone go undetected, leading to a diagnostic ‘gray zone’ in which standard markers are insufficient to distinguish malignant from non-clonal cytopenias. This review highlights the critical added value of integrating functional biomarkers, specifically the nuclear proliferation marker Ki-67 and the anti-apoptotic protein Bcl-2, into the diagnostic and prognostic workflow. The results of studies using these two biomarkers illustrate the need to redefine biological behavior, in addition to the immunophenotype of the malignant clone, to better capture the dynamic pathobiology of MDS. Shifting the focus from “cell phenotype” to “cell behavior” marks a paradigm shift in the management of myeloid malignancies.

## 1. Introduction

Myelodysplastic syndromes (MDS), represent a heterogeneous group of clonal hematopoietic stem cell disorders [1,2] defined by ineffective hematopoiesis, dysplasia in one or more myeloid cell lines, and a variable risk of transformation into secondary acute myeloid leukemia (sAML) [3,4,5,6,7,8,9]. A defining clinico-pathological hallmark is the “MDS paradox”: the coexistence of a hypercellular bone marrow (BM) with peripheral cytopenias [3,6,10,11]. Although driven by complex mutational landscapes, this phenomenon results largely from a critical imbalance between cell cycle progression and (excessive) programmed cell death [3,7,12]. Understanding how these opposing biological forces operate within a malignant cell clone is central to both the diagnosis and the rational selection of therapy.

**Figure 1:**
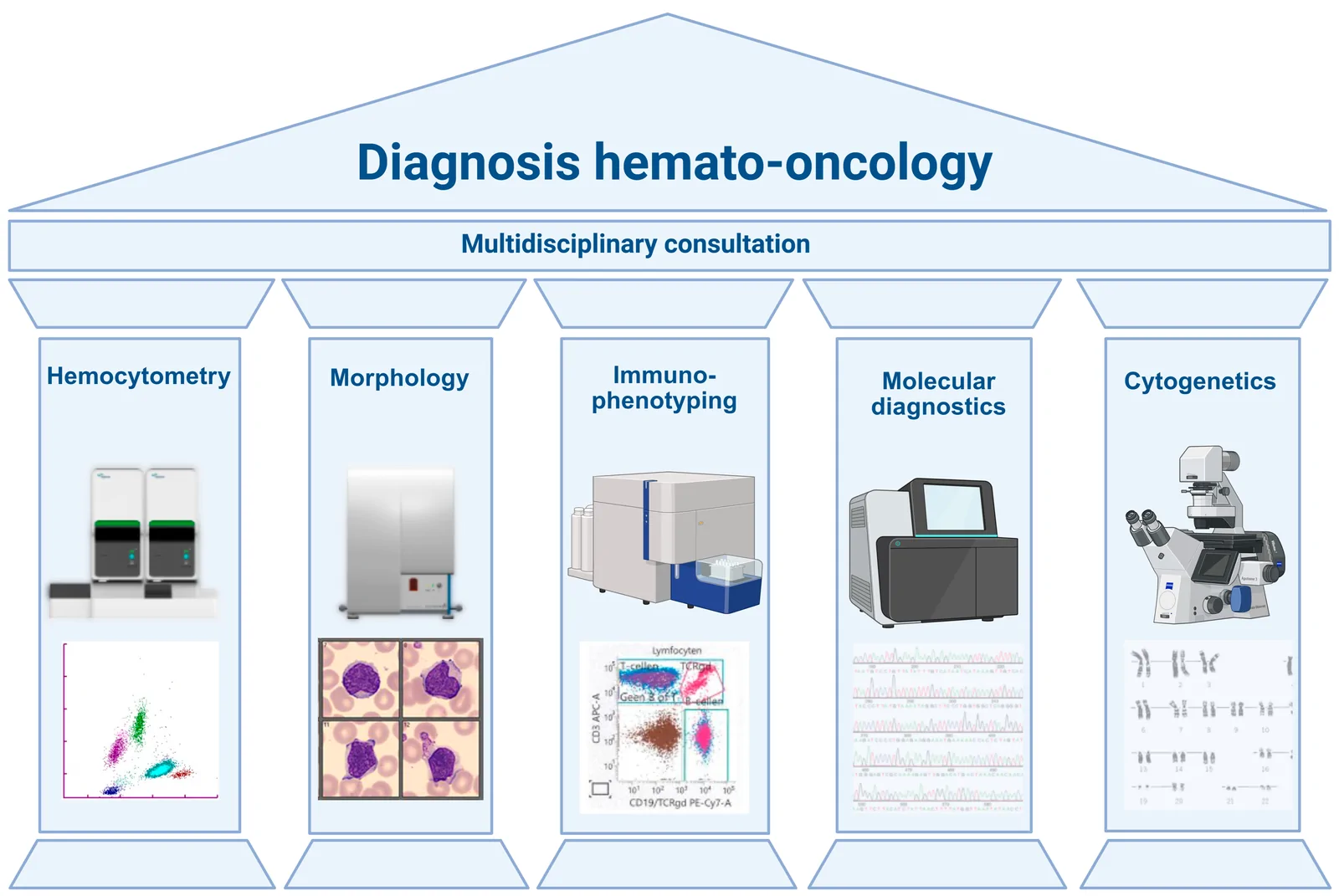
The current diagnostic workflow for myeloid malignancies involves a multidisciplinary approach integrating hemocytometric findings, peripheral blood and BM morphology, blast counts, flow cytometric immunophenotyping, molecular diagnostics (including next-generation sequencing [NGS]), and cytogenetics.

The current diagnostic workflow, as defined by the WHO 2022 and the International Consensus Classification (ICC) [13,14], emphasizes a multidisciplinary integration of hemocytometry findings, morphology, immunophenotyping, molecular diagnostics (including next-generation sequencing [NGS]), and cytogenetics (Figure 1) [15,16,17,18]. The introduction of the Molecular International Prognostic Scoring System (IPSS-M) has further refined risk stratification by incorporating somatic mutations in genes such as SF3B1, ASXL1, and TP53 as disease-defining parameters [1,2,16]. Despite these advancements, morphological evaluation remains the gold standard. However, its inherent subjectivity leads to significant inter-observer variability, particularly in the diagnostic ‘gray zone’ of low-grade MDS [19,20,21].

While this review focuses on the synergy between proliferation (Ki-67) and anti-apoptosis (Bcl-2), several other functional approaches can complement the characterization of the malignant clone [22]. For instance, assessing oxidative stress (by measuring intracellular reactive oxygen species; ROS) and metabolic status (e.g., mitochondrial membrane potential or lysosomal activity) provides insights into cellular survival mechanisms and homeostasis. Additionally, investigating signaling pathways through phospho-flow cytometry [23] and performing ex vivo chemosensitivity assays (e.g., testing response to Venetoclax) [24] can further identify therapeutic vulnerabilities. Integrating these alternative functional biomarkers alongside Ki-67 and Bcl-2 may offer a broader framework for precision medicine in MDS and AML.

Multiparameter flow cytometry (MFC) has become an essential ancillary tool for lineage assignment and the detection of aberrant immunophenotypes [2,5,8,25,26]. Despite the objectivity of flow cytometric analysis, it is widely regarded as highly dependent on expertise and as having limited reproducibility in multicenter studies. This perception likely relates to the increasing number of lasers, detectors, antibodies, and fluorochromes used, and the corresponding increase in the complexity of multivariate data analysis for both major and minor cell populations. Additionally, limited standardization of laboratory procedures and instrument settings is a factor. Recent efforts by the European LeukemiaNet (ELN), International MDS-Flow Cytometry Working Group (iMDSFlow), and the EuroFlow Consortium have led to the development of guidelines for flow cytometry standardization, sample handling, and data analysis. In addition, these consortia identified a core set of 17 immunophenotypic markers independently associated with MDS diagnosis. Despite this, a correct methodological framework is essential for proper multiparameter functional flow cytometry (MFFC) [20,27,28]. In Section 2, we will take a closer look at these kinds of analytical issues. Yet, established systems like the Ogata score and the Integrated Flow Score (iFS), rely exclusively on static phenotypic markers, defining only cell phenotypes rather than their biological behavior at a specific time point [5,21,25,29]. Consequently, these scores often show limited sensitivity (54–75%) in low-grade cases lacking increased blasts or ringed sideroblasts, precisely where objective biomarkers are most needed [5,19,20,21,27,30].

We propose that integrating dynamic functional biomarkers alongside conventional immunophenotyping can overcome these limitations by capturing the biological behavior of the malignant clone. Two such indicators are the nuclear proliferation marker Ki-67 and the anti-apoptotic protein Bcl-2. Ki-67 serves as the gold standard for measuring proliferative activity across all active cell-cycle phases, while Bcl-2 is a central regulator of the mitochondrial apoptotic pathway; its overexpression protects the malignant clone from undergoing apoptosis and is a major mechanism of therapy resistance [31,32].

In Section 3, we will discuss the proliferative aspect of MDS and AML, with a focus on Ki-67 expression. Our recent studies have redefined the functional landscape of MDS, identifying ineffective proliferation as a central paradox of the disease [33,34,35]. We observed a significant decrease in cell-cycle progression during early maturation stages, most prominently in the erythroid lineage, where the Ki-67 index dropped from a normal level of approximately 70% to about 40%. By integrating the erythroid Ki-67 proliferation index as a fifth parameter into the conventional Ogata score, diagnostic sensitivity for low-grade MDS increased from 56% to 91% while maintaining 100% specificity [36]. Furthermore, a reduced erythroid Ki-67 index (≤28%) has emerged as a powerful independent predictor of transfusion dependency within 1 year, even in patients with mildly reduced hemoglobin levels at diagnosis [37].

In Section 4, we take a closer look at the counterpart to proliferation, namely the apoptotic process, as determined by the quantification of Bcl-2, an anti-apoptotic marker. Beyond diagnostics, the Bcl-2:Ki-67 ratio provides a superior metric of biological aggressiveness, directly influencing treatment decisions. A high ratio indicates a quiescent yet apoptosis-resistant cell population, whereas a low ratio indicates a cell population with relatively high proliferative activity. This may influence the therapeutic choices and prognosis, underscoring its clinical relevance for personalized management [35,38].

This review discusses how functional flow cytometry bridges the gap between complex pathobiology and precision medicine, making a necessary shift toward individualized management in myeloid malignancies.

## 2. Methodological Framework: Multiparameter Functional Flow Cytometry (MFFC)

Substantial technological advancements in MFFC have driven the transition from traditional, static immunophenotyping to high-dimensional functional cell characterization. While early studies [39,40,41] faced limitations, recent validation efforts demonstrate that multi-color flow cytometry reliably quantifies dynamic indicators such as proliferation and apoptosis, ensuring reproducibility across laboratories [33,38,42].

The successful implementation of MFFC for detecting rare cell populations in the BM requires rigorous standardization of the pre-analytical phase, which can be challenging in certain clinical settings. Variations in sample preparation may affect cell yield, viability, and phenotypic accuracy, highlighting the need for standardized protocols to ensure consistent results [38,43,44].

### 2.1. Standardized Sample Processing and Functional Immunostaining

High-dimensional functional characterization of functional biomarkers requires rigorous standardization of sample handling to ensure antigen stability and reproducible metrics. Consensus guidelines recommend processing BM aspirates within 48 to 72 h of retrieval to maintain antigen stability [28,41].

In addition, when detecting and quantifying both cell-surface-exposed differentiation markers and intracellular functional biomarkers, several critical methodological aspects must be considered.

#### 2.1.1. Pre-Analytical Quality

Anticoagulation: A primary concern during BM sample collection is coagulation, as clots can disrupt the homogeneity of the cell suspension and lead to inaccurate measurements [40]. To counteract this process, anticoagulants are essential. Standard agents include heparin, EDTA, and acid–citrate–dextrose [40]. Pre-filling/rinsing aspiration needles and syringes with a heparin solution before collection is a common procedure to prevent early clot formation [39,40,42,44]. While heparin effectively prevents clotting, its interactions with cellular biology are complex, as it may facilitate cell proliferation and, at high concentrations, interfere with specific trilineage differentiation in long-term cultures [44].

Hemodilution: Beyond clotting, contamination with peripheral blood (hemodilution) poses a significant risk to BM sample quality [40,44,45]. To minimize peripheral blood influx and ensure a high concentration of relevant progenitor cells, technical studies recommend using small aspiration volumes (1-4 mL) per site and smaller syringes (e.g., 10 mL) [39,44,46]. Smaller syringes generate stronger negative pressure with less frictional resistance, resulting in progenitor cell concentrations that are on average three times higher than those obtained using larger 50 mL syringes [44]. An artificial increase in the total nucleated cell count due to hemodilution can lead to a drastic underestimation of the actual fraction of rare (abnormal) cells in the BM [40,44,45].

Enzymatic digestion: A significant improvement to the methodological framework is the application of enzymatic digestion as a pre-treatment step for BM material [45,47,48]. Several technical studies emphasize that a substantial portion of resident BM cell populations is tightly anchored to the extracellular matrix or trabecular bone, making them difficult to retrieve by simple aspiration alone [38,49]. Standard flushing techniques may fail to liberate up to 96% of matrix-bound cells [38]. The use of enzymes such as collagenase or dispase to break down the matrix in bone fragments or trephine biopsies can dramatically increase cell recovery [38,43,47,49,50]. Technical comparisons show that enzymatic extraction can yield a 65- to 100-fold increase in the yield of rare cells compared to conventional aspirates [45,49]. This method is particularly valuable in clinical “dry tap” situations where liquid BM cannot be easily aspirated [48,51]. Cells liberated through enzymatic digestion remain phenotypically identical to their aspirated counterparts, providing a more representative and abundant population for detailed MFFC analysis [45,49].

Mechanical dissociation: Beyond enzymatic digestion, mechanical dissociation represents a significant and standardized alternative for liberating cells from rigid bone marrow fragments or biopsies. Automated systems, such as the MediMachine (DAKO/CTSV), use disposable capsules containing rotating stainless-steel blades or filters to physically reduce tissue into a single-cell suspension. This method minimizes operator-dependent variability and enhances reproducibility compared to manual techniques like manual mincing or scraping with scalpel blades [52]. Another effective mechanical approach is vortex dissociation, where tissue is briefly but vigorously agitated in a culture medium; this technique is particularly valuable for small biopsies and inaspirable bone marrow specimens (“dry taps”) [53]. A major advantage of mechanical dissociation is the speed of the procedure (often less than one minute) and the ability to keep the antigenic epitope almost fully intact. Although mechanical methods drastically reduce processing time compared to enzymatic digestion, and avoid certain regulatory restrictions associated with enzymatic methods, thorough validation is required: physical shear forces can increase cellular debris and reduce cell viability.

Bulk lysis technique: Recent evidence suggests that for high-sensitivity detection of rare malignant populations, such as CD34+ progenitors and CD117+ mast cells [54], a bulk-lysis procedure (e.g., using ammonium chloride) is superior to conventional lysing methods, enabling the acquisition of ≥10^7^ cells per sample [29].

Enrichment of specific cell populations: Processing the resulting suspension often involves additional steps, such as erythrocyte lysis and cell enrichment using antibody-conjugated magnetic beads (e.g., via CD271+ selection or CD45/Ter119 depletion) to enable the detection of extremely rare cell sub-populations [38,39,43,55]. While these enrichment steps are necessary for functional assays, they may entail significant cell loss during centrifugation and washing [42]. Studies report median losses of approximately 35% of viable cells during initial centrifugation steps [42]. Consequently, it is critical to monitor cell viability (using dyes such as 7-AAD or DAPI) and absolute cell counts throughout the processing pipeline to ensure reliable MFFC results [38,39,43,55]. Finally, the lack of fully standardized international protocols for isolation and pre-treatment remains a challenge, highlighting the need for consensus to ensure comparability of FCM data across different laboratories [44,46,56].

#### 2.1.2. Immunostaining Phase

Simultaneous cell surface and cytoplasmic/intranuclear immunostaining: Following surface staining, cells can undergo a specialized fixation and permeabilization treatment [33,35,57,58]. This is a prerequisite for penetration by antibodies targeting the nuclear proliferation marker Ki-67 (e.g., monoclonal antibody MIB-1) and the mitochondrial anti-apoptotic protein Bcl-2 [35,58,59,60]. The permeabilization step can be performed by using organic solvents such as methanol or buffers containing detergents such as Tween 20 or saponin [61]. Specific buffer sets are commercially available that allow sequential staining of cell-surface antigens and intracellular markers while preserving cell morphology and scatter characteristics [62].

A significant technical challenge in the quantification of Ki-67 and Bcl-2 may be the relatively low fluorescence intensity when using commercially available conjugated antibodies, which can introduce subjectivity when positioning positive analysis regions. Unlike many lineage-specific surface markers that exhibit distinct bimodal distributions, the fluorescence emission of these functional biomarkers often presents a continuum emerging directly from the negative cell population. Consequently, there is typically no clear gap between negative and positive cells, which could potentially weaken the harmonization of staining methods in multicenter studies. To minimize operator-dependent variability and ensure reproducibility, we have previously conducted extensive methodological evaluations to test various gating strategies [59,60]. These studies allowed us to establish an optimized and standardized gating procedure that more accurately defines the positive Ki-67 and Bcl-2 fractions. By utilizing these refined settings, it is possible to achieve a more objective readout of the malignant clone’s behavior, even in the absence of a clear-cut negative-to-positive transition.

Gating strategies, from isotype controls to FMO: Accurate measurement of antigen-positive cell populations is often hampered by background fluorescence, which primarily arises from three sources: autofluorescence, spectral overlap, and undesirable antibody binding [63]. While isotype-matched controls have traditionally been used to estimate non-specific binding, they are increasingly recognized as problematic in complex multicolor panels. A critical limitation of isotype controls is their inability to account for ‘spreading error’, an increase in the variance of the negative population caused by the spectral compensation of other fluorochromes in the panel mix. Consequently, the Fluorescence-Minus-One (FMO) approach is now the widely accepted standard for defining positive gating regions. By including all antibodies in the panel except the one being tested, FMO controls provide a precise baseline that incorporates both background binding and the specific spreading error associated with the complete staining cocktail [63]. In our previous functional studies, we further optimized this by utilizing a backbone of five identical markers in every tube of the panel. This strategy serves a dual purpose: it acts as a partial FMO to effectively correct for background staining, while simultaneously providing common anchor points to allow the merging and calculation of multi-tube datasets into a single, high-dimensional biological fingerprint. This methodology, supported by expert recommendations, ensures a more objective and reproducible characterization of malignant clones in patient samples [33,35,36,57,58].

### 2.2. Advanced Data Analysis

One of the key challenges in high-dimensional MFFC is the inability to measure all relevant markers simultaneously within a single tube due to technical limitations in fluorochrome and detector availability. Therefore, data acquired from multiple tubes must be computationally integrated. To overcome this, the “Merge and Calculate” procedure (e.g., available within the Infinicyt v2.0 software) can be applied [35,57,58,59]. This strategy fuses multiple data files from the same patient into a single, multidimensionally merged file. By applying the nearest-neighbor principle, the software calculates missing values, providing a complete functional and phenotypic profile for every individual cell [18,57,58,64]. This enables holistic biological fingerprinting that analyzes numerous immunophenotypic features alongside their biological behavior.

Such an analysis moves even further beyond discrete gating by evaluating cells along a maturation continuum [58,65]. Using software-based tools, the differentiation pathways of the erythroid, myeloid, and monocytic lineages can be divided into discrete immunophenotypic stages [34,35,58,65]. This innovation enables the detection of subtle functional aberrations at distinct maturation stages [34,35,65]. Recent studies have validated this approach, showing that analyzing maturation trajectories, often visualized via FlowSOM unsupervised clustering, is more sensitive than traditional counts for identifying dysplastic subsets [66].

### 2.3. Establishment of Age-Matched Reference Values

Biological processes such as proliferation and apoptosis undergo age-related alterations, while clinical interpretation must rely on well-matched reference datasets [58,65]. Indicative reference ranges have been established for Ki-67 and Bcl-2 indices across all hematopoietic lineages in BM samples from healthy, older, age-matched patients, derived from femoral hip specimens after replacement [35,58,65]. These reference values serve as a baseline for objectively identifying the functional derailment that characterizes MDS and its progression toward AML [35,57,58,65].

By integrating these advanced methodological pillars, functional flow cytometry provides a robust, objective, and reproducible window into the survival and growth kinetics that define the pathobiology of MDS.

## 3. Ki-67 as a Metric for Proliferative Inefficiency in MDS

Quantitative assessment of cell proliferation is a cornerstone of diagnostic histopathology, in which the nuclear antigen Ki-67 serves as the gold-standard biomarker for tumor grading and clinical prognosis [67]. Expressed during all active phases of the cell cycle (late G1, S, G2, and M) but notably absent in quiescent (G0) cells, Ki-67 provides a direct measure of the growth fraction of a cell population [68,69,70]. While its utility is well-established in solid tumors and lymphomas, its integration into the diagnostic flow cytometric workflow for myeloid malignancies remained sparse for decades, largely due to technical limitations of early 2- to 4-color flow cytometry, and conflicting kinetic evidence regarding the nature of BM hypercellularity [33,36,68].

### 3.1. Resolving the MDS Paradox

A defining clinico-pathological hallmark of MDS, as redesignated by the WHO 2022 classification [14], is the MDS paradox, resulting in a hypercellular BM but lack of mature peripheral cells [71]. This phenomenon has traditionally been attributed to a disturbance in the balance between cell cycle progression and excessive programmed cell death [72].

Early investigations into the proliferative activity of MDS yielded conflicting results. Studies using markers such as proliferating cell nuclear antigen (PCNA) or bromodeoxyuridine incorporation into replicating DNA suggested that early-stage MDS was characterized by abnormally increased progenitor proliferation as a compensatory mechanism for peripheral cytopenias [3,73]. Conversely, other flow cytometric or autoradiographic methods reported a progressive reduction in the rate of cell cycle progression [74].

A seminal study by Jensen et al. [75] addressed these discrepancies by utilizing multiparameter flow cytometry to simultaneously evaluate markers for immature myeloid cells (using CD13 and CD33) and DNA content. They demonstrated a clear decrease in the proliferative activity of immature myeloid cells as the disease progressed, revealing a significant negative correlation between the fraction of myeloblasts and cell cycle activity. These findings suggested that the differentiation block in MDS is fundamentally coupled with a simultaneous block in proliferation, particularly in more advanced stages. This critical observation provides a biological basis for the hypercellular bone marrow paradox, identifying proliferative inefficiency as a central feature of MDS [34,35].

### 3.2. Proliferative Inefficiency in Early Maturation Stages

Building upon the seminal observation of Jensen et al., our research confirms that MDS is fundamentally defined by proliferative inefficiency, a significant reduction in the growth fraction during early maturation across all assessed lineages [35]. This biological phenomenon provides the mechanical explanation for the hypercellular BM paradox: the marrow is densely packed with dysfunctional cells that fail to successfully divide and mature.

The most pronounced derailment occurs within the erythroid lineage, which is morphologically dysplastic in approximately 88% of patients with MDS [76]. While the Ki-67 index in healthy early erythroid progenitors is approximately 70%, it drops significantly to approximately 40% in patients with MDS [35,37]. This reduction is a hallmark of ineffective erythropoiesis, and is often accompanied by the relative accumulation of immature CD117+ erythroid progenitors [35,57,77].

Critically, this functional profile allows for the definitive distinction of MDS from reactive, compensatory bone marrow responses. Unlike non-clonal cytopenias (e.g., nutrient deficiencies or acute blood loss), which typically trigger a regenerative drive with an increased Ki-67 index, MDS is uniquely characterized by a maturation block despite the hypercellular environment. Our previous studies [33,35,58,60,65] confirmed that this special functional profile, particularly when measured within the erythroid lineage, allows for the distinction of MDS from reactive states with high specificity.

### 3.3. Diagnostic Improvement by Combining the Ogata and Ki-67 Scores

The practical clinical value of measuring this proliferative inefficiency is most evident in the diagnosis of low-grade MDS. These patients often reside in a diagnostic gray zone, with potential premalignant MDS conditions, including idiopathic cytopenias of undetermined significance (ICUS) or clonal hematopoiesis with indeterminate potential (CHIP), in which morphological dysplasia is subjective and objective markers, such as increased blast percentages or ring sideroblasts, are absent [78,79,80]. Established flow cytometric tools, such as the four-parameter Ogata score, primarily utilize static biomarkers (e.g., size of the CD34+ myeloblast fraction, CD34+ B-cell progenitor percentage, blast/lymphocyte CD45 ratio, and granulocyte side scatter) [19,79]. While highly specific for the diagnosis of MDS (92-98%), the Ogata score demonstrates limited sensitivity, frequently failing to detect low-grade MDS in 30% to 44% of cases [30,79,81].

Our research has demonstrated that the erythroid Ki-67 proliferation index (using a diagnostic cut-off of ≤37%) is the strongest independent parameter for distinguishing MDS from non-clonal cytopenias [36]. By integrating this functional marker as a fifth parameter into the Ogata score, diagnostic sensitivity for low-grade MDS increased from 56% to 91% while maintaining 100% specificity [36]. This refined scoring system captures the biological essence of these neoplasms, i.e., ineffective hematopoiesis including the most affected lineage, facilitating early and accurate intervention.

### 3.4. Prognostic Value: Predicting Transfusion Dependency

Anemia is the most common presenting feature in MDS, affecting 80–90% of symptomatic patients. The onset of red blood cell transfusion dependency is linked to diminished quality of life and reduced overall survival. Although baseline hemoglobin levels are a central parameter of cytopenia in the Revised International Prognostic Scoring System (IPSS-R), serving as a surrogate for BM failure, these values may not accurately reflect real-time erythropoietic activity due to the cumulative effects of erythrocyte lifespan and transfusion support [82,83,84,85].

In addition to its diagnostic utility, the Ki-67 proliferation index serves as a robust prognostic indicator that augments established systems, including the International Prognostic Scoring System (IPSS) for MDS [1]. Previous studies have demonstrated that total bone marrow proliferation indices, such as PCNA or Ki-67 (MIB-1) labeling, predict overall survival, especially in high-risk MDS subgroups [3].

Our lineage-specific investigations have demonstrated that a reduced erythroid Ki-67 proliferation index (≤28%) at initial diagnosis is a strong, independent predictor for the development of transfusion dependency within one year [37]. This functional biomarker enables the identification of high-risk individuals, including those with only mildly reduced baseline hemoglobin levels. By reflecting the actual biological production of cells in the BM, the Ki-67 proliferation index provides an essential early-warning indicator for clinical management and the timely initiation of erythropoiesis-stimulating agents (ESAs) or other targeted therapies [86]. The prognostic threshold of ≤28% for transfusion dependency was established through Receiver Operating Characteristic (ROC) curve analysis within our study cohort [37]. Although this threshold showed a strong correlation with clinical outcomes in our dataset, it should be noted that validation in an independent, external cohort is still ongoing. Further prospective studies are required to confirm the reproducibility and broad clinical applicability of this specific cut-off across different laboratory settings.

## 4. Bcl-2 and the Bcl-2:Ki-67 Ratio: Survival Versus Growth in Myeloid Malignancies

The dynamic interplay between cell-survival mechanisms and proliferative capacity fundamentally determines the clinical outcomes and biological progression of myeloid malignancies. While current diagnostic protocols, such as those established by EuroFlow [18,25,29,64,87,88], primarily rely on static immunophenotypes for lineage assignment, recent studies emphasize that a shift toward functional biomarkers provides a more nuanced understanding of how malignant clones achieve medullary dominance. Central to this biological behavior is the anti-apoptotic protein Bcl-2 and its relationship with the proliferation marker Ki-67, forming a “survival versus growth” paradigm that is critical for characterizing MDS [35,57].

### 4.1. The Biological Shift: From Apoptosis to Clonal Survival

As mentioned before, the pathobiology of early-stage MDS (e.g., MDS with low blast cell counts) is defined by a paradoxical state: a hypercellular BM that fails to produce mature cells due to ineffective hematopoiesis and excessive intramedullary apoptosis [6,19,72,89,90]. In these low-grade stages, apoptosis significantly outpaces proliferation, often with an apoptosis-to-proliferation ratio of >2 [72,73].

However, disease progression is marked by a fundamental biological pivot [11]. As MDS progresses to advanced stages (MDS with increased blast cell counts) and eventually to sAML, the apoptotic fraction decreases while the expression of anti-apoptotic proteins, most notably Bcl-2, increases [7,31,91].

Bcl-2 functions as a survival factor by inhibiting the mitochondrial pathway of programmed cell death, thereby protecting aberrant cells from both endogenous death signals and chemotherapeutic insults [7,31,92,93,94]. Historically, high Bcl-2 expression in myeloid precursors has been a strong predictor of transformation to AML and reduced response to intensive chemotherapy, as standard cytotoxic regimens primarily target actively dividing cells and depend on the induction of apoptosis [91,95]. Campos et al. [96] established that, for the same reason, high Bcl-2 levels in AML blasts are associated with a reduced response to intensive chemotherapy, a finding confirmed in more recent studies [31,32,96,97].

### 4.2. Niche-Induced Survival: The Role of the BM Microenvironment

Recent evidence underscores that Bcl-2 upregulation is not merely an intrinsic cellular event but is actively modulated by the BM microenvironment [7,98]. Specialized CXCL12+ stromal cells create a protective niche for CD34+ hematopoietic cells [98]. In MDS and AML, the density of these CXCL12+ cells is significantly higher than in normal BM and correlates positively with the BM blast ratio. Through CXCL12/CXCR4 signaling, these stromal cells induce Bcl-2 upregulation in the malignant clone, creating an environment that promotes anti-apoptotic effects and clonal survival [98]. Triple immunostaining has revealed that CD34+ cells in direct contact with CXCL12+ cells are consistently Bcl-2 positive and TUNEL (Terminal deoxynucleotidyl transferase dUTP Nick End Labeling)-negative, suggesting they are resistant to the apoptotic signals prevalent in the BM of MDS patients [98]. This niche-mediated survival mechanism explains why clusters of immature precursors (also called Abnormal Localization of Immature Precursors; ALIP) often localize near CXCL12+ reticular cells, facilitating disease progression and chemoresistance.

### 4.3. The Bcl-2:Ki-67 Ratio as a Metric of Biological Aggressiveness and Prediction of Therapy Response

The concurrent quantification of Bcl-2 anti-apoptotic activity and Ki-67 proliferative activity enables the integration of the two markers into a single Bcl-2:Ki-67 ratio, providing a powerful indicator of a disturbed balance between survival and growth [35,57].

To strengthen the clinical value of the Bcl-2:Ki-67 ratio as a metric of biological aggressiveness, orientative numerical ranges can be used. In the immature CD34+ progenitor compartment, patients with MDS with low blast cells (MDS-LB) typically exhibit ratios between 1 and 5. Upon progression to MDS with an increased frequency of blast cells (MDS-IB1 and MDS-IB2) and eventually AML, this ratio significantly escalates, frequently exceeding 10 and reaching values above 100 in advanced cases (ref). A biological ‘grey zone’ is identified between a ratio of 5 and 10, where overlap between low-grade and high-grade stages occurs, reflecting the phase where the malignant clone acquires a dominant survival advantage. While this ratio is most informative within the blast population, the monopoietic lineage consistently demonstrates the highest ratios across all MDS stages.

**Figure 2:**
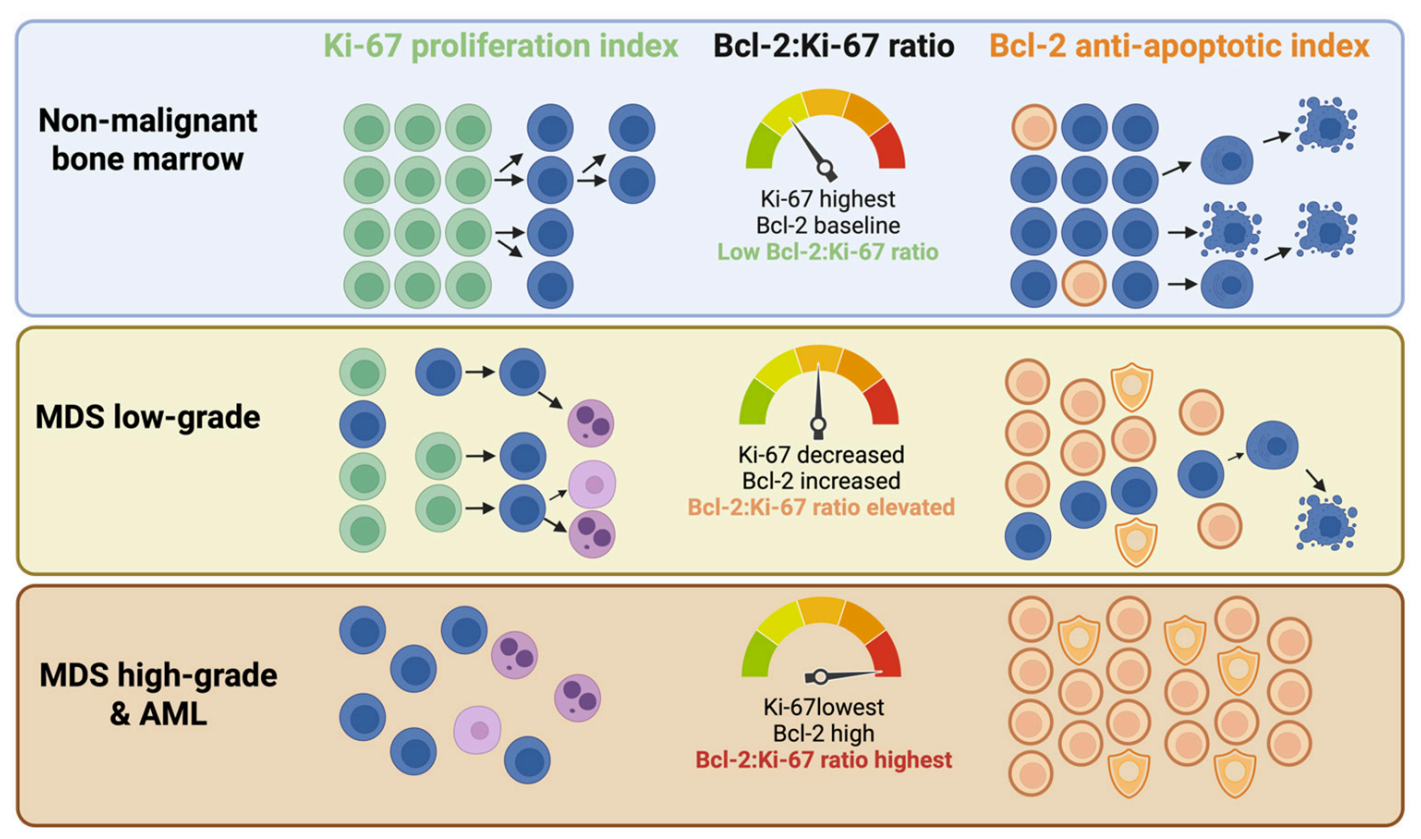
MDS can be functionally understood through three interrelated axes: proliferation, apoptosis-resistance, and their balance. This figure shows a schematic representation of Ki-67- and Bcl-2-positive cell populations in the BM of healthy individuals and patients suffering from MDS and AML. A low Bcl-2:Ki-67 ratio indicates that proliferation predominates over anti-apoptotic signaling, whereas a high Bcl-2:Ki-67 ratio indicates a quiescent but apoptosis-resistant cell population.

The practical impact of these functional markers may lie in individualizing therapy selection based on biological behavior. On the one hand, a low Bcl-2:Ki-67 ratio indicates that proliferation predominates over anti-apoptotic mechanisms, and, as a result, these cells are likely to be sensitive to traditional cytotoxic chemotherapy [35,57]. On the other hand, a high Bcl-2:Ki-67 ratio identifies a quiescent but apoptosis-resistant population. These cells are typically refractory to standard chemotherapy but may be highly vulnerable to targeted inhibition of Bcl-2 (Figure 2) [35,57].

Cell types indicated in different colors: Ki-67 proliferation index panel: green: proliferating immature BM cells; blue: non-proliferating immature BM cells; pink: mature non-dividing cells.

Bcl-2 anti-apoptotic index panel: blue: healthy immature BM cells; orange: immature BM that are protected against apoptosis; blue fragmented cells: apoptotic BM cells; Shield cells: malignant immature cells that are protected against apoptosis and arrested in their maturation. Note also cell fragmentation, indicating cells are undergoing apoptosis.

The introduction of the Bcl-2 inhibitor Venetoclax, often in combination with hypomethylating agents (HMAs), has transformed the management of high-risk MDS [4]. Recent retrospective real-world data indicate that this combination achieves an overall response rate of 75% in patients with high-risk MDS, significantly higher than the rates reported for HMAs alone [4]. By quantifying these intertwined processes of proliferation and survival using the Bcl-2:Ki-67 ratio, clinicians may move beyond phenotypic description to a direct assessment of therapeutic targets, thereby optimizing efficacy and reducing treatment-related mortality in patients with myeloid malignancies and paving the way for truly personalized medicine in MDS.

Another quantitative measure for the expression levels of proliferative and (anti-)apoptotic compounds is their Median Fluorescence Intensity (MFI). Such flow cytometry parameters have been described as more robust markers for predicting response to Venetoclax than mRNA expression levels [99,100]. Furthermore, combining MFI measurements for Bcl-2, Bcl-xL, and Mcl-1 significantly improves the predictive power of these biomarkers [101]. While Mcl-1 is a known driver of Venetoclax resistance, Azacitidine has been shown to reduce Mcl-1 levels, potentially explaining the synergy between the two agents [97,101]. Additionally, the percentage of BCL2L10 (Bcl-B, or Bcl-2 like 10)-positive cells has emerged as an independent prognostic factor for the outcome of Azacitidine treatment, with high levels (>10%) correlating with resistance and shorter overall survival [100].

## 5. Future Perspectives

The clinical framework established in Section 3 and Section 4 rests on a rapidly evolving methodological foundation, shifting from a diagnosis based primarily on subjective morphology, complemented by static immunophenotyping, to a multimodal integration of genomics, digital imaging, and high-dimensional functional analysis [5,8]. The future of MDS management lies in a deeper understanding of the malignant clone’s biological behavior through analysis of functional biomarkers. Advanced data analysis of results from such biomarker tests may, in the future, contribute significantly to more objective diagnosis and prognosis in MDS cases and to predicting therapeutic efficacy in these patients.

In MFFC, unsupervised clustering tools such as FlowSOM enable detection of subtle immunophenotypic aberrations that are invisible to the human eye [8]. To enable multicenter utility, the measurement of Median Fluorescence Intensity (MFI) must be strictly standardized [25]. The use of P-score metrics and Rainbow eight-peak beads enables rapid identification of technical outliers, ensuring that biological differences in Bcl-2 or Ki-67 expression are not masked by instrument variation [25].

Studies using random forest classifiers have shown that MDS can be diagnosed with processing times under two minutes while maintaining high objectivity and sensitivity [66]. Our goal is to develop a fully integrated “intelligent” diagnostic workflow in which AI harmonizes clinical, laboratory, and functional flow cytometric data to provide a unified risk score [16].

### 5.1. The Integration of Computational Flow Cytometry

The inherent subjectivity of manual gating and blast enumeration is increasingly being addressed through artificial intelligence (AI) and machine learning (ML) [16]. This transition is necessitated by the rising complexity of high-dimensional datasets, now reaching 40+ colors and millions of events, which exceed the cognitive capacity of manual evaluation [25,102,103]. Computational workflows utilizing unsupervised clustering (e.g., FlowSom) and machine learning classifiers (such as Random Forrest) can now process these complex panels in under two minutes, achieving a sensitivity of up to 97% and a specificity of 95% [102,104,105,106].

To ensure clinical reliability, these tools are underpinned by automated preprocessing pipelines, such as Peak Extraction and Cleaning Oriented Quality Control (PeacoQC) and FlowAI, which standardize data cleaning and doublet and debris removal [102,103,104,107,108,109]. Beyond automation, computational flow cytometry (CFC) offers an unbiased approach to identifying novel pathobiological features invisible to the human eye, such as increased side scatter in maturing erythroid cells, a highly discriminative hallmark of dyserythropoiesis [104]. Furthermore, CFC enables precise mapping of maturation trajectories and the identification of rare, aberrant hematopoietic stem and progenitor cell subsets, such as the CD34+CD38- population, which are critical indicators of leukemic transformation [110,111].

By integrating functional biomarkers into these workflows, we can achieve high-resolution quantification of Ki-67 and Bcl-2 indices across discrete immunophenotypic stages [34,105]. Our ultimate goal is a fully integrated diagnostic workflow where AI harmonizes static immunophenotypes, clinical parameters, and functional readouts, such as the Bcl-2:Ki-67 ratio, into a unified, actionable risk score. Such a multimodal approach would not only refine the diagnosis within the gray zone of cytopenias of undetermined significance (such as ICUS or CCUS), and, in the case of Clonal Hematopoiesis of Indeterminate Potential (CHIP), but also provide a biological read-out to guide personalized therapeutic interventions, such as targeted Bcl-2 inhibition [104]. These data-driven approaches are already yielding superior prognostic tools, such as the FCM-prognostic score (FCM-PS) [112], which has demonstrated an improved ability to predict overall survival compared to the traditional IPSS-R [112], by integrating independent parameters like mature B-lymphocyte and plasmacytoid dendritic cell counts.

### 5.2. Tailored Therapy: The Use of Functional Biomarkers in Precision Medicine

The clinical value of functional biomarkers primarily lies in the individualization of therapeutic choices. While conventional chemotherapy targets rapidly dividing cells, malignant blasts in MDS are often quiescent yet highly resistant to apoptosis. The Bcl-2:Ki-67 ratio serves as a superior metric for this “survival without growth” phenotype [35,57]. This ratio allows clinicians to distinguish between patients likely to be sensitive to cytotoxic agents (having a low Bcl-2:Ki-67 ratio) and those with a resting, apoptosis-resistant clone that is refractory to standard regimens (having a high ratio).

The introduction of the Bcl-2 inhibitor Venetoclax, combined with HMAs, has transformed the treatment of high-risk MDS, achieving response rates of up to 75% [4] Our data indicate that a functional protein readout is superior to Bcl-2 mRNA measurements, as protein abundance is the actual determinant of sensitivity to Venetoclax [99,101].

While the Bcl-2:Ki-67 ratio shows promise as a metric of biological behavior, direct comparative analysis with established diagnostic and prognostic scales, such as the Ogata-score or IPSS-M, is required in future studies to fully define its independent clinical value and warrant integration into routine practice.

In addition, at present, no clinical trials have utilized this ratio as a predefined primary or secondary endpoint. The current evidence for its clinical utility is based on retrospective datasets and biological characterization. Consequently, prospective clinical trials are required to formally validate this functional ratio as a predictive marker for therapy response in MDS and AML.

Furthermore, functional erythroid markers are proving vital in the management of lower-risk MDS. Quantification of CD117+ erythroid precursors and the erythroid Ki-67 proliferation index can now be used to predict which patients will respond to erythropoiesis-stimulating agents (ESAs) or novel therapies such as Luspatercept [113].

A critical advantage of this approach is its logistical and economic feasibility: it utilizes existing hardware and expertise within hematology-oncology departments, with only a marginal increase in reagent costs. This ensures that these dynamic biological markers can be readily incorporated into the routine budget and workflow of modern hematology laboratories.

Finally, while BM examination remains the mandatory gold standard, there is a strong shift toward developing non-invasive technology [5,114]. Digital computational analysis of peripheral blood smears and detection of somatic mutations from peripheral blood genetics are promising avenues for investigation [5,115]. The detection of circulating CD34+ hematopoietic stem cells via single-cell RNA sequencing may eventually allow for a peripheral blood-based diagnosis, potentially sparing many patients from invasive and painful BM aspiration procedures [115]. In this respect, it is meaningful to investigate whether detecting Ki-67 and Bcl-2 levels in relevant cell populations from peripheral blood samples can aid in the minimally invasive diagnosis of patients with MDS.

## 6. Conclusions: A Paradigm Shift in MDS Management

The transition from identifying only phenotypic characteristics to also identifying the biological behavior of the malignant clone may lead to a fundamental paradigm shift in the routine clinical practice of myeloid malignancies. Recent studies underscore the critical added value of integrating functional biomarkers into the diagnostic and prognostic workflow for MDS.

The clinical impact of this functional approach is established across three pillars:

1. Enhanceddiagnostic accuracy: Integrating the Ki-67 proliferation index into established scores like the Ogata score increases sensitivity for low-grade MDS, effectively bridging the gap in morphologically indistinct cases.
2. Robust prognostic prediction: A reduced erythroid Ki-67 proliferation index serves as a powerful independent predictor of transfusion dependency within one year, facilitating proactive clinical management.
3. Targeted therapy selection: The high Bcl-2:Ki-67 ratio identifies an aggressive phenotype, accurately flagging patients for Venetoclax-based therapies and providing a biological read-out of resistance mechanisms.

By adopting standardized protocols, there is an ongoing need for more efficient fluorochromes and optimized staining protocols to further enhance the quality of Bcl-2 and Ki-67 signal detection. This technical improvement, combined with the application of age-matched reference values, will allow for the precise measurement of survival and growth kinetics. Furthermore, while the Bcl-2:Ki-67 ratio provides a robust biological readout of the ‘survival versus growth’ kinetics, its transition from retrospective characterization to prospective clinical validation is now required. Future clinical trials that utilize this functional ratio as a predefined primary of secondary endpoint will be essential to definitively establish its clinical utility in guiding therapeutic interventions, such as targeted Bcl-2 inhibition. Only through such rigorous validation can these dynamic functional metrics become a standardized component of precision medicine in MDS and AML [16].

## Data Availability

No new data were created or analyzed in this study. Data sharing does not apply to this article.

## Abbreviations

The following abbreviations are used in this manuscript:

7-AAD	7-Amino-actinomycine D
AI	Artificial intelligence
AML	Acute myeloid leukemia
BM	Bone marrow
BrdU	Bromodeoxyuridine
CCUS	Clonal cytopenia of undetermined significance
CD	Cluster of Differentiation
CFC	Computational flow cytometry
CHiP	Clonal hematopoiesis with indeterminate potential
cMRD	Computational measurable residual disease
DAPI	4′,6-Diamidino-2-phenylindole
ELN	European LeukemiaNet
FMO	Fluorescence-Minus-One
ICC	International Consensus Classification
ICUS	Idiopathic cytopenia of uncertain significance
iFS	Integrated flow-score
HMAs	Hypomethylating agents
iMDS Flow	International Myelodysplastic Syndrome Flow Cytometry Working Group
IPSS-M	Molecular International Prognosis Scoring System
MDS	Myelodysplastic syndrome
MDS-IB	Myelodysplastic syndrome with increased blasts
MFC	Multiparameter flow cytometry
MFFC	Multiparameter functional flow cytometry
MRD	Minimal residual disease
PCNA	Proliferating cell nuclear antigen
ROC	Receiver Operator Characteristics
sAML	Secondary acute myeloid leukemia
WHO	World Health Organization
